# Synthesis and In-Vivo Evaluation of Benzoxazole Derivatives as Promising Anti-Psoriatic Drugs for Clinical Use

**DOI:** 10.3390/molecules27093023

**Published:** 2022-05-08

**Authors:** Rami Ayoub, Jamal Jilani, Qais Jarrar, Raad Alani, Chrismawan Ardianto, Khang Wen Goh, Dalia Ali, Said Moshawih

**Affiliations:** 1 Department of Applied Pharmaceutical Sciences and Clinical Pharmacy, Faculty of Pharmacy, Isra University, Amman 11622, Jordan; qais.jarrar@iu.edu.jo; 2 Department of Medicinal Chemistry and Pharmacognosy, Faculty of Pharmacy, Jordan University of Science and Technology, Irbid 22110, Jordan; jilanij@just.edu.jo; 3 Department of Physiotherapy, Faculty of Allied Medical Sciences, Isra University, Amman 11622, Jordan; raad.alani@iu.edu.jo (R.A.); dalia.ali@iu.edu.jo (D.A.); 4 Department of Pharmacy Practice, Faculty of Pharmacy, Universitas Airlangga, Surabaya 60115, Indonesia; 5 Faculty of Data Science and Information Technology, INTI International University, Nilai 71800, Malaysia; 6 PAP Rashidah Sa’adatul Bolkiah Institute of Health Sciences, Universiti Brunei Darussalam, Gadong BE1410, Brunei; saeedmomo@hotmail.com

**Keywords:** synthesis, arylbenzoxazole, psoriasis, imiquimod, in vivo, prodrug

## Abstract

2-(4-Chlorophenyl)-5-benzoxazoleacetic acid (CBA) and its ester, methyl-2-(4-chloro-phenyl)-5-benzoxazoleacetate (MCBA), were synthesized, and their structures were confirmed by ^1^HNMR, IR, and mass spectrophotometry. The anti-psoriatic activities of CBA and MCBA were tested using an imiquimod (IMQ)-induced psoriatic mouse model, in which mice were treated both topically (1% *w/w*) and orally (125 mg/kg) for 14 days. The erythema intensity, thickness, and desquamation of psoriasis were scored by calculating the psoriasis area severity index (PASI). The study also included the determination of histopathological alterations in the skin tissues of treated mice. Topical and oral administration of CBA and MCBA led to a reduction in erythema intensity, thickness, and desquamation, which was demonstrated by a significant decrease in the PASI value. In addition, skin tissues of mice treated with CBA and MCBA showed less evidence of psoriatic alterations, such as hyperkeratosis, parakeratosis, scale crust, edema, psoriasiform, and hyperplasia. After administration of either topical or oral dosing, the anti-psoriatic effects were found to be stronger in MCBA-treated than in CBA-treated mice. These effects were comparable to those produced by Clobetasol propionate, the reference drug. This drug discovery could be translated into a potential new drug for future clinical use in psoriasis treatment.

## 1. Introduction

Psoriasis is an immune-mediated disease that has a persistent effect on skin tissues. It is a non-communicable disease that affects around 0.84% of the global population [1]. Patients with psoriasis exhibit several skin abnormalities, such as hyperproliferation, atypical keratinocytes, epidermal hyperplasia, inflammation, leukocyte infiltration, and increased vascularity in the skin layers [2,3]. The diagnostic features of psoriasis include skin redness, thickness, and silver scaly plaques that may cover a focal or wide area of the elbows, knees, scalp, and lumbar region [4]. These features are frequently associated with itching, scaling, burning, erythema, and bleeding [5]. The pathophysiology of psoriasis is related to complex interactions between various immune, cellular, and molecular factors, in addition to the genetic and protein expression of various inflammatory mediators, such as interleukins (IL)- 6, -17, -22, and -23, interferon-gamma (IFNγ), nuclear factor kappa B (Nf-κB), and tumor necrosis factor-alpha (TNF⍺), as described by Croxford et al. [6], Ha et al. [7], Lowes, Suarez-Farinas, and Krueger et al. [8], and Raphael et al. [9]. From a clinical point of view, psoriasis symptoms are frequently managed by convenient drugs, such as corticosteroids, and immunosuppressive agents, such as methotrexate, vitamin D analogs, and systemic antiproliferative agents, in addition to biological agents, such as infliximab and etanercept [10,11,12]. However, the therapeutic outcomes of these drugs often result in unsatisfactory progress and may be associated with serious adverse effects, including nausea, swelling, headaches, hair growth, and others [3,13,14]. Therefore, the synthesis and development of novel drugs are necessary to match the current clinical demand for psoriasis treatment and management. Benzoxazole and its derivatives are N-heterocyclic aromatic compounds found in various natural sources, such as fungi, marine life, and plant origins, and medicines, such as flunoxaprofen and benoxaprofen [15]. During the last several decades, benzoxazole and its derivatives have gained a lot of importance because of their extensive biological properties, such as anticancer [16], analgesic [17], antimicrobial [18], neuroprotective [19], inflammatory bowel disease [20], and immunosuppressive [21] activities. According to a previous study, chronic treatment with oral benoxaprofen, a benzoxazole derivative, produced excellent anti-psoriatic effects in human patients, which may partially be attributed to its capability to inhibit epidermal 5-lipoxygenase activity or inhibit the accumulation of phagocytes in psoriatic lesions [22]. Accordingly, the current study was conducted to synthesize two benzoxazole derivatives and evaluate their anti-psoriatic activity using imiquimod-induced psoriasis-like dermatitis in mice.

## 2. Results

### 2.1. Synthesis

**Scheme 1:**
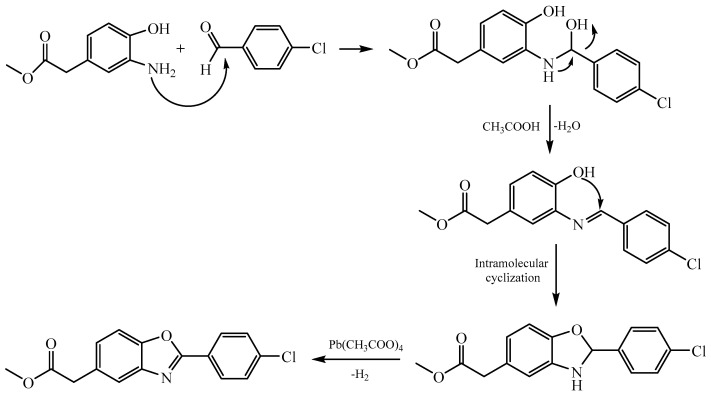
Synthesis of methyl 2-(4-chlorophenyl) benzoxazol-5-yl) acetate (MCBA).

The synthesis of MCBA was achieved by oxidative coupling of chlorobenzaldehyde with ortho aminophenol in the presence of lead tetraacetate as an oxidizing agent, as Scheme 1 shows. The starting materials, methyl 2-(3-amino-4-hydroxyphenyl)acetate and 4-chlorobenzaldehyde, were obtained from commercially available sources.

**Scheme 2:**
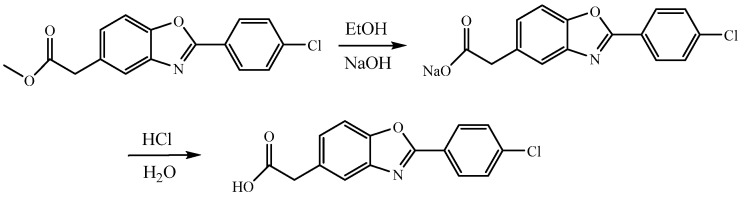
Synthesis of 2-(4-chlorophenyl)-5-bnenzoxazoleacetic acid (CBA).

On the other hand, free carboxylic benzoxazole, CBA, was obtained by the hydrolysis of MCBA, as the Scheme 2 below shows:

### 2.2. Effects of Topical IMQ on Mouse Skin

**Figure 1:**
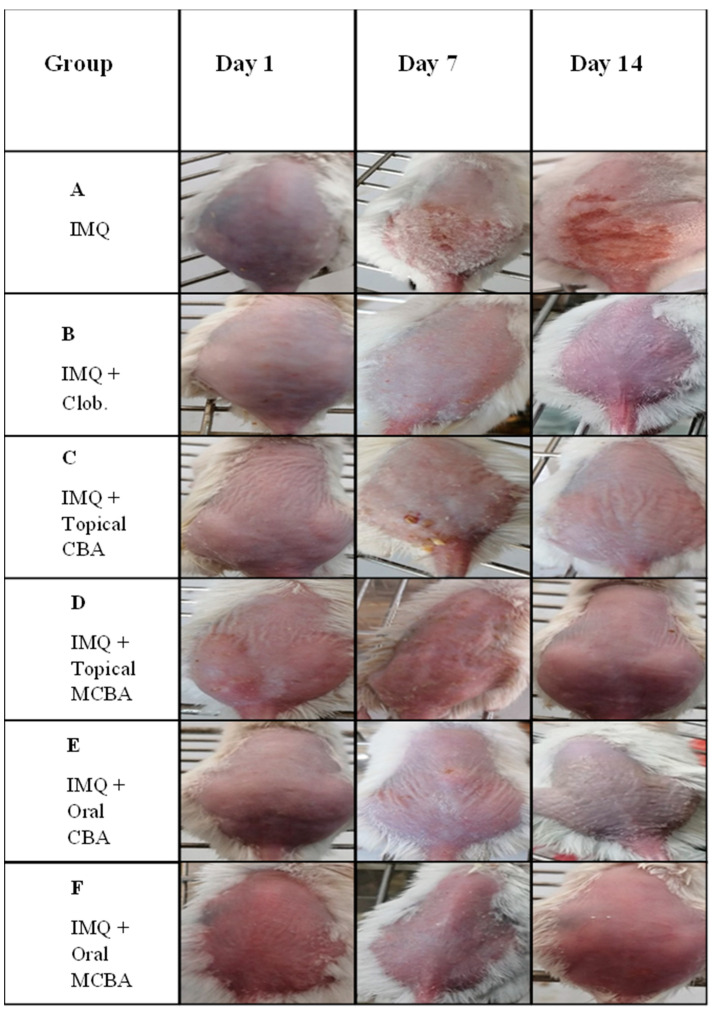
Representative gross skin features in mice of different groups on days 1, 7, and 14. (**A**) IMQ-treated mice. (**B**) Clobetasol propionate (Clob)-treated mice. (**C**) Topical CBA-treated mice. (**D**) Topical MCBA-treated mice. (**E**) Oral CBA-treated mice. (**F**) Oral MCBA-treated mice.

**Figure 2:**
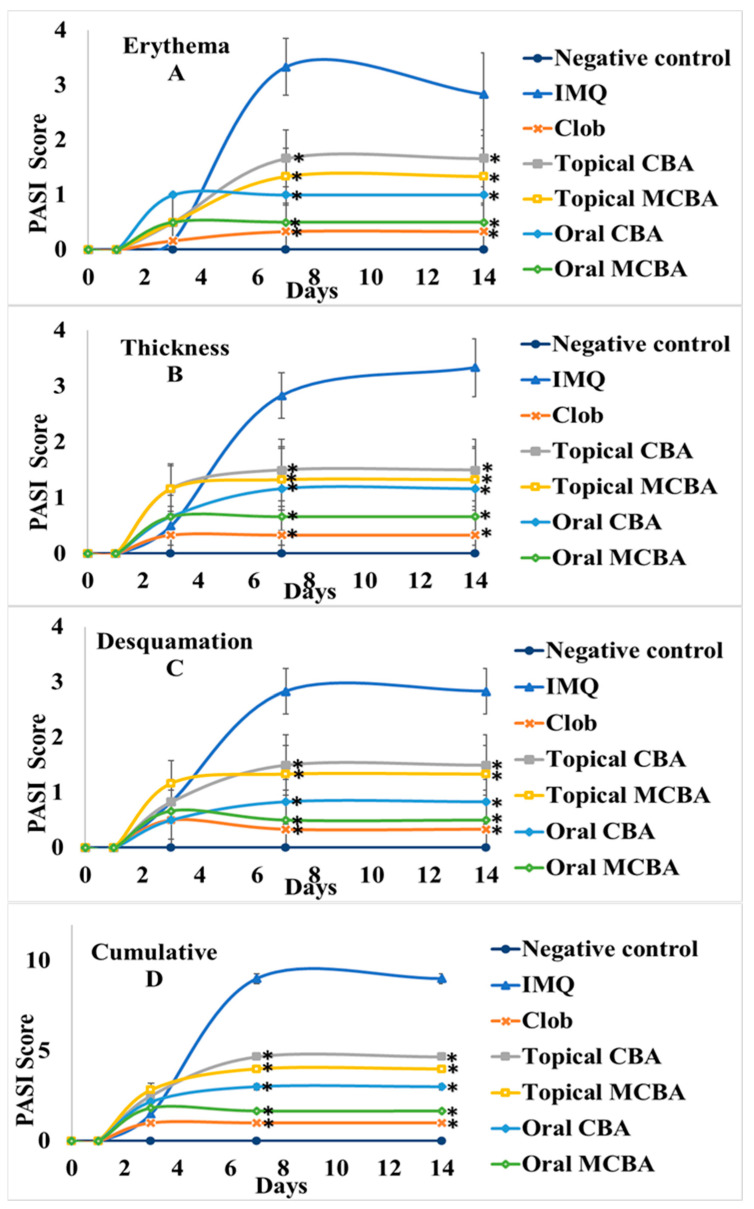
Psoriasis area and severity index (PASI) scores of different groups on days 1, 3, 7, and 14. (**A**) Erythema score, (**B**) thickness score, (**C**) desquamation score and, (**D**) cumulative score. (*) Indicates significant difference (*p* < 0.05) from IMQ-treated group (*n* = 6).

**Figure 3:**
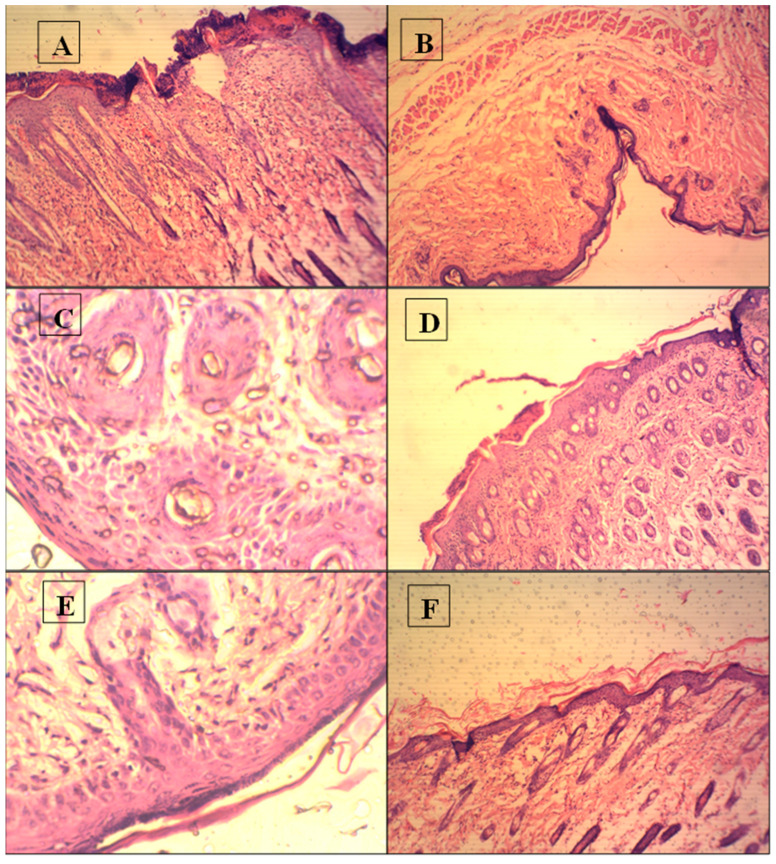
Representative photomicrographs of skin in mice of different groups. (**A**) IMQ-treated mice showing hyperkeratosis, parakeratosis, scale crust, edema, psoriasiform, and hyperplasia. (**B**) clobetasol-treated mice showing orthokeratosis. (**C**) Topical MCBA-treated mice showing hyperkeratosis, parakeratosis, scale crust, hyper granulosis, intact epidermal–dermal junction, focal psoriasiform epithelium, few polymorphs in upper dermis, few extravasates red blood cells, and scattered apoptosis cells in epidermis. (**D**) Topical CBA-treated mice showing mild hyperkeratosis, parakeratosis, scale crust, edema in upper dermis, neutrophils in upper dermis, and focal psoriasiform hyperplasia. (**E**) Oral MCBA-treated mice showing focal mild hyperkeratosis, focal parakeratosis, focal hyper granulosis, and intact epidermal–dermal junction. (**F**) Oral CBA-treated mice showing orthokeratosis, subcutaneous tissue showing pus cells, and necrosis of hypodermis.

Three days after starting IMQ treatment, it was observed that the dorsal skin of the treated mice exhibited signs of erythema, thickness, and desquamation compared to the dorsal skin of the negative control group. The phenotypic effects of IMQ are demonstrated in Figure 1A. The maximum erythema score was observed on day 7 with an average of 3.3 (Figure 2A). On the other hand, signs of thickness and desquamation were observed from days 7 to 14 with average scores of 3.3 and 2.8, respectively (Figure 2B and C, respectively). Examination of the histological slides showed that the skin tissues of mice treated with IMQ exhibited acanthosis (thickening of the stratum spinosum layer), hyperkeratosis, and neutrophil infiltration into the epidermis, in addition to subcutaneous hemorrhage and inflammatory cell infiltration in the dermis around sweat glands and between connective tissue and muscles (Figure 3A). The capillaries of the upper and middle portions of the dermis were dilated.

### 2.3. Effects of Clobetasol and Benzoxazole Derivatives on Dermatitis-Like Psoriasis

Gross observations showed that treatment with Clob in addition to MCBA and CBA via either the topical or oral route caused a marked reduction in dermatitis-like psoriasis (Figure 1B–F), which was determined by a significant decrease in PASI erythema scores (Figure 2A), thickness (Figure 2B), desquamation (Figure 2C), and cumulative score (Figure 2D). Histopathological examination showed that the dorsal skin of mice treated with Clob showed a marked decrease in inflammatory infiltration and hyperplasia of the epidermis (Figure 3B). Topical application of CBA and MCBA caused the partial suppression of IMQ-induced epidermal hyperplasia and a reduction in the number of infiltrated inflammatory cells (Figure 3C,D). On the other hand, oral treatment with CBA and MCBA caused anti-psoriatic effects comparable to Clob, which was manifested by a marked decrease in hyperkeratosis, parakeratosis, hypergranulosis, and psoriasiform hyperplasia (Figure 3E,F). However, the anti-psoriatic effects were more evident in groups treated with MCBA than those treated with CBA.

## 3. Discussion

### 3.1. Chemistry

In this study, CBA and MCBA were developed and synthesized as anti-psoriatic agents. According to the literature, several synthetic methods are available to prepare benzoxazole derivatives [23]. One of these synthetic processes involves the coupling reaction of benzaldehyde with an o-aminophenol derivative using lead tetraacetate as an oxidizing agent [16,20]. Some research groups prepared a series of arylbenzoxazoleacetic acid derivatives, including our target CBA, using ethyl 4-chlorobenzimidate hydrochlorides and 3-amino-4-hydroxyphenylacetic acid [24,25]. In this work, we preferred to prepare our target compounds by coupling 4-chlorobenzaldehyde with methyl-3-amino-4-hydroxyphenylacetic acid in the presence of lead tetraacetate. Schiff bases or benzoxazole compounds represent attractive molecular scaffolds of enormous medicinal and industrial relevance. The Schiff base condensation reaction was obtained from the nucleophilic attack of the amine group in methyl 2-(3-amino-4-hydroxyphenyl)acetate with the electrophilic carbon atom of aldehyde of 4-chlorobenzaldehyde to form carbinolamine. After that, under acidic conditions using CH_3_COOH, a Schiff base was formed by water elimination. Finally, the intermediate underwent intramolecular cyclization, followed by Pb(CH_3_COO)_4_ for oxidation, leading to the formation of MCBA. On the other hand, CBA was synthesized by the hydrolysis of MCBA ester utilizing a mild and rapid procedure with the use of ethanol and sodium hydroxide. The use of this method produced CBA salt, which was acidified using HCl, leading to the formation of CBA. The chemical structures of MCBA and CBA were confirmed by ^1^H NMR, FTIR, and mass spectrophotometry. ^1^H NMR of CBA showed a singlet at δ 3.73 ppm for the benzylic protons, while in the aromatic region, the spectra showed a doublet at δ 7.3 with a *J* value of 8.5 Hz, which represents proton #7, a multiplet at δ 7.56–7.59 ppm for three protons, a singlet at δ 7.63 for one proton, and a doublet at δ 8.164-8.181, which represents two protons with a *J* value of 8.5 Hz. On the other hand, ^1^H NMR for MCBA showed almost the same spectra as CBA, except for an additional singlet at δ 3.67 ppm for the methyl ester protons. Concerning FTIR spectra of CBA, it showed characteristic peaks for carbonyl at 1668.85 cm^−1^ and a broad band at 2828.27 cm^−1^ for the OH group. On the other hand, the methyl ester MCBA exhibited a characteristic peak for the ester carbonyl at 1733.29 cm^−1^ in addition to a stretching peak at 1260.32 cm^−1^ for C-O, which was absent in the acid CBA. The mass spectrum for MCBA showed peaks at m/z 302 for the molecular ion [M+H]+. In addition, the MCBA spectrum showed a base peak at m/z 230, which represents MCBA after losing the methylacetate side chain. The mass spectra for CBA showed peaks at m/z 288 for the molecular ion [M+H]+. The product yield was relatively sufficient, indicating the suitability of this method for the synthesis of both CBA and MCBA for further experimental applications.

### 3.2. Anti-Psoriatic Activity

Psoriasis is an autoimmune disease and is typically diagnosed based on physical (macroscopic) and microscopic examinations [26]. Gross skin alterations include signs of erythema, desquamation, and thickness [27]. On the other hand, histopathological alterations of psoriasis include parakeratosis, a diminished or nonexistent granular layer, papillomatosis, acanthosis, chronic inflammation, dilatation of capillaries, edema, and abscess (collection of neutrophils) in the stratum corneum [28]. For experimental evolution, topical IMQ application on the dorsal back skin of the mice has been used as a valid model for the induction of dermatitis-like psoriasis. This model has been frequently used for screening possible anti-psoriatic drugs [29]. Despite clinical progress in the treatment of various diseases, no effective and safe drug for the treatment of psoriasis has been found. Systemic steroids have been frequently used as the treatment of choice for the management of psoriasis, but these drugs are often associated with several clinical limitations [27]. From the pharmacological point of view, the anti-psoriatic effects of steroidal drugs have been shown to relapse after discontinuation of the treatment [30]. In addition, tolerance occurs with long-term use of these medications, as patients no longer respond to the treatment, and escalating doses are needed to maintain the therapeutic effects [31]. Treatment with these drugs is also associated with dermatological and systemic adverse effects, such as skin atrophy, striae, acne, weight gain, water retention, swelling, diabetes, and osteoporosis [32]. Together, these adverse effects suggest the need for a novel drug regimen that possesses greater therapeutic benefits and fewer adverse effects. In this study, a benzoxazole derivative, CBA, and its prodrug, MCBA, were synthesized and evaluated as promising drugs for psoriasis treatment. The benzoxazole family group of chemicals exert a wide range of pharmacodynamics with potent immunosuppressive and anti-inflammatory effects [33]. One line of evidence demonstrated that benzoxazole derivatives have immune-modulatory effects on T-lymphocyte activity that contribute to the pathogenesis of psoriasis [21]. On the other hand, prodrugs have been used as effective drug carriers for enhancing therapeutic activity and reducing drug toxicity [34]. A prodrug is an inactive substance that undergoes metabolic or physicochemical changes in the body that produce a pharmacologically active agent [35]. The main function of prodrugs is to enhance pharmacokinetics and deliver drugs in sufficient concentrations to the target site of action [36]. The findings of the current work showed that both oral and topical administration of CBA and MCBA produced considerable anti-psoriatic effects, which were demonstrated by a significant decrease in PASI scores and histopathological alterations as compared to those of IMQ-treated mice. These effects were more pronounced in animals that were administered oral doses compared to those given topical treatments. In addition, MCBA produced a stronger inhibitory effect on various parameters in the mice than CBA. Together, these results indicate that both MCBA and CBA are effective drugs for psoriasis treatment and management. The increase in drug activity after oral administration compared to those of topical treatments could be due to improved pharmacokinetics, thus allowing for stronger molecular effects at the target site of action. In addition, MCBA showed a stronger anti-psoriatic effect, which could be partially attributed to its enhanced lipophilicity, which may lead to an increase in systemic bioavailability.

## 4. Materials and Methods

### 4.1. Chemicals

Chemicals of synthetic grade were used. The chemicals were purchased from various companies: (1) Methyl 2-(3-amino-4-hydroxyphenyl)acetate from Abcr (Karlsruhe, Germany); (2) 4-chlorobenzaldehyde, ethanol, dimethylsulfoxide (DMSO), and hydrochloric acid (HCl) from Acros (Geel, Belgium); (3) glacial acetic acid, lead tetraacetate, and sodium hydroxide from Sigma (St. Louis, MO, USA); (4) Clobetasol propionate (Clob) from GSK (London, UK); and (5) imiquimod (IMQ) cream (Aldara) from Meda (Bad Homburg, Germany).

### 4.2. Instruments

Gas chromatography/mass spectrometry (GC/MS) real-time analysis software from Craig S Young. Sr. Technical Support (Shimadzu Scientific Instruments, Tokyo, Japan) was used. Fourier transform infrared (FT-IR) spectra (from 500 to 4000 cm^−1^) of the products were recorded using a Thermo Nicolet 670 Nexus NFT-IR spectrophotometer from Thermo Scientific (Massachusetts, MA, USA). ^1^H-NMR spectra were recorded on a Bruker 500 MHz-Avance III (Billerica, Massachusetts, MA, USA). A microtome (Anglia Scientific 0325 Instruments, Cambridge, England), microscope (Nikon, Tokyo, Japan), and Fujisan digital caliper (Fujisan, Japan) were used in this study.

### 4.3. Synthesis of Methyl 2-(2-(4-Chlorophenyl)benzo[d]oxazol-5-yl)acetate (MCBA)

Methyl 2-(3-amino-4-hydroxyphenyl)acetate (2.0 g, 0.011 mol) was dissolved in 15 mL of absolute ethanol in a round-bottom flask with a capacity of 50 mL, and 4-chlorobenzaldehyde (1.54 g, 0.011 mol) was then added. The reaction mixture was stirred with continuous reflux for 4 h. The resulting mixture was evaporated under reduced pressure to obtain viscous material that was then dissolved in a suitable volume (15–20 mL) of hot glacial CH_3_COOH, treated directly with Pb(CH_3_COO)_4_ (5.7 g, 0.013 mol), and stirred until it reached room temperature (25 °C). The resulting crystalline solid was filtered on cellulose filter paper, washed with distilled water, recrystallized with ethanol, and then dried to give 2.3 g of MCBA; yield 70%; m.p.140–144 °C; FTIR (cm^−1^): 1733.29 cm^−1^ (C=O), 1260.32 cm^−1^ (C-O). ^1^H-NMR (CD_3_CN), δ ppm 3.67 (s, 3H, OCH_3_), 3.78 (s, 2H, benzylic H), 7.318–7.335 (d, 1H, *J* = 8.5 Hz, ArH), 7.59–7.62 (m, 3H, ArH), 7.65 (s, 1H, ArH), 8.186–8.203 (d, 2H, *J* = 8.5 Hz, ArH). MS (ESI) m/z: Calcd for C_16_H_13_ClNO_3_ [M+H]+: 302.0505. Found: 302.0514.

### 4.4. Synthesis of 2-(4-Chlorophenyl) benzoxazol-5-yl) acetic acid ( CBA)

Synthesis of CBA was performed via hydrolysis of MCBA (1.0 g, 0.0034 mol), which was dissolved in 90% ethanol (50 mL), treated with NaOH (0.6 g,0.015 mol), and stirred at room temperature (25 °C) for 3 h. The ethanol was then evaporated, and the residue was collected and poured into a mixture of crushed ice (20 mL) and concentrated HCl (2 mL). A white solid crystal precipitated and was filtered with a Buchner funnel and then recrystallized with ethanol to give 0.75 g solid product; 79% yield; m.p. 182–185 °C; FTIR (cm^−1^): 1668.85 (C=O acid), 2828.27 cm^−1^ (OH). ^1^HNMR (CD_3_CN) δ ppm: 3.73 (s, 2H, benzylic H), 7.29–7.31 (d, 1H, *J* = 8.5Hz, ArH), 7.56–7.59 (m, 3H, ArH), 7.63 (s, 1H, ArH), 8.164–8.181 (d, 2H, *J* = 8.5 Hz, ArH). MS (ESI) *m/z*: Calcd for C_15_H_11_ClNO_3_ [M+H]+: 288.0349. Found: 288.0352.

### 4.5. Animal Husbandry and Care

Male BALB/c mice of the same age (6–8 weeks) were used in the current work as the model of choice for the evaluation of psoriasis. Mice were obtained from Isra University, kept under controlled temperature (20 ± 2 °C) and humidity (60 ± 5 °C), and given free access to tap water and food pellets. All animal manipulation and handling were conducted according to the Guide for the Care and the Use of Laboratory Animals [37]. The animal use protocol was approved by the Scientific Research Ethics Committee of Isra University (SREC/21/12/018).

### 4.6. Animal Groups and Treatments

Mice were separated into seven groups (*n* = 6 mice/group); one was a negative control group that did not receive any treatments, while the other six groups were given different treatments, as shown in Table 1. The procedure for induction of psoriasis was obtained from previous studies [29]. In brief, the back dorsal skin of mice was shaved, and topical IMQ cream was then applied daily (5% *w*/*w* at a dose of 62.5 mg) for 14 days.

### 4.7. Scoring the Severity of Psoriasis-like Dermatitis

The intensity of erythema, thickness, and desquamation of the affected dorsal skin of mice was scored independently by calculating PASI on a 4-point scale (0 indicated no observed change, 1 indicated slight change, 2 indicated moderate change, 3 indicated marked change, and 4 indicated severe change). The cumulative scores (erythema plus scaling plus thickness) were used to determine the severity of skin inflammation, which ranged from 0 to 12. Skin thickness measured by digital caliper, in addition to erythema and desquamation, was observed daily over the 14 days of the study.

### 4.8. Histological Examinations

Preparation of histological slides was performed according to a previously described procedure (Pang et al., 2018) [38]. After animals were sacrificed with an overdose inhalation of diethyl ether, skin tissue samples were removed and fixed in 10% neutral-buffered formaldehyde. The samples were then dehydrated with ethanol, cleared with xylene, and embedded in paraffin blocks. The paraffin sections were cut to 5 μm thick and stained with hematoxylin and eosin before being observed under a light microscope.

### 4.9. Statistical Analysis

Data obtained from PASI scoring analysis are presented as mean ±standard deviation (S.D.). GraphPad Prism software, version 8, was used to assess significant differences between the experimental groups using one-way analysis of variance (ANOVA) followed by Tukey’s post hoc test for multiple comparisons. A statistically significant difference was defined as a *p* < 0.05.

## 5. Conclusions

Based on the findings of the current work, it can be concluded that benzoxazole derivatives are promising candidates for causing a reduction in inflammatory and psoriatic symptoms in mice with psoriasis-like dermatitis. However, developing these compounds as prodrugs may present great potential to enhance their anti-psoriatic effects. Further studies are needed to assess the toxicity, pharmacokinetics, and molecular mechanisms of their pharmacological actions in preclinical experiments before performing clinical investigations.

## Figures and Tables

**Table 1:** Experimental groups in anti-psoriatic activity experiments.

Group Number	Group Type	Description
Group I	Negative control	Each mouse did not receive any treatment
Group II	Model control	Each mouse received a daily dose (62.5 mg) of 5% IMQ cream that was topically applied on the shaved dorsal skin
Group III	Positive control	Each mouse received a daily dose (62.5 mg) of 5% IMQ cream that was topically applied on the shaved dorsal skin + topical administration of Clob 0.05% cream
Group IV	Topical MCBA	Each mouse received a daily dose (62.5 mg) of 5% IMQ cream that was topically applied on the shaved dorsal skin + topical administration of 1% MCBA
Group V	Topical CBA	Each mouse received a daily dose (62.5 mg) of 5% IMQ cream that was topically applied on the shaved dorsal skin + topical administration of 1% CBA
Group VI	Oral MCBA	Each mouse received a daily dose (62.5 mg) of 5% IMQ cream that was topically applied on the shaved dorsal skin + oral administration of MCBA (120 mg/kg)
Group VII	Oral CBA	Each mouse received a daily dose (62.5 mg) of 5% IMQ cream that was topically applied on the shaved dorsal skin + oral administration of CBA (120 mg/kg)

## Data Availability

The data presented in this study are available on request from the corresponding author.

## Sample Availability

Samples of the compounds are available from the authors.
